# Prevalence and genomic analysis of t203-like G9 (G9-VI) rotaviruses circulating in children with gastroenteritis in Beijing, China

**DOI:** 10.1007/s00705-023-05860-0

**Published:** 2023-09-27

**Authors:** Hui-Jin Dong, Li-Ying Liu, Li-Ping Jia, Lin-Qing Zhao, Feng-Hua Jin, Lin Zhou, Yuan Qian

**Affiliations:** 1 Laboratory of Virology, Beijing Key Laboratory of Etiology of Viral Diseases in Children, Capital Institute of Pediatrics, Beijing, 100020 China; 2 Department of Infectious Diseases, Affiliated Children’s Hospital to Capital Institute of Pediatrics, Beijing, 100020 China; 3 Department of Clinical Laboratory, Affiliated Children’s Hospital to Capital Institute of Pediatrics, Beijing, 100020 China

## Abstract

Our previous surveillance revealed that t203-like G9 (tentatively designated subtype G9-VI) rotaviruses re-emerged in 2010 in Beijing and rapidly prevailed over the G9-III subtype (the most common G9 subtype globally) and previously predominant G genotypes over the following two years. G9-VI belongs to the VP7 evolutionary lineage VI, which includes unusual and sporadic human rotaviruses from China (t203) and Japan. To obtain insight into the epidemiology, evolution, and transmission advantages of G9-VI rotavirus, we performed follow-up surveillance (2014-2017) and whole-genome analysis of 12 representative G9 strains. The results showed that the G9 genotype was predominant (77.4%), with a marked increase in prevalence (previously 43.5%). Within the G9 genotype, subtype G9-VI accounted for the majority (98.3%) of cases. The most prevalent P-genotype was P[8] (93.7%), within which subtype P[8]b was rare (0.7%). Phylogenetically, the G9-VI subtype strains in this study clustered closely with contemporary emerging human rotaviruses from many other countries in VP7 lineage VI, indicating that this subtype is capable of spreading globally. These currently emerging G9-VI rotaviruses formed a distinct monophyletic subcluster when compared to early G9-VI rotaviruses. Furthermore, four specific amino acid substitutions and synonymous codon substitutions were observed in the VP7 genes between the current G9-VI and globally common G9-III rotaviruses. The remaining nine genes of all of the analyzed representative G9 strains, whether G9-VI or G9-III, combined with the P[8]a, P[8]b, or P[6] genotype and exhibited the same Wa-like backbone constellation.

**Supplementary Information:**

The online version contains supplementary material available at 10.1007/s00705-023-05860-0.

## Introduction

Group A rotaviruses (RVAs), which belong to the family *Sedoreoviridae*, are the leading cause of severe acute gastroenteritis in infants and young children, as well as in many animal species [1]. Globally, RVA strains were responsible for an estimated 128,500 deaths of children under the age of five in 2016 [2]. The highest rates of rotavirus mortality and hospitalization occur in low-income countries, particularly in sub-Saharan Africa and South Asia [2, 3].

The RVA genome consists of 11 double-stranded (ds) RNA gene segments, which encode six structural (VP1–VP4, VP6, and VP7) and six nonstructural (NSP1–NSP5/6) proteins [1]. RVAs are commonly classified using a binary classification system based on the genes encoding the proteins VP7 (glycoprotein, G) and VP4 (protease-sensitive protein, P), both of which are outer capsid proteins that independently evoke neutralizing antibodies [1]. To date, 41 G and 57 P genotypes have been reported in humans and animals [4]. Of these, G1-G4, G9, and G12 and P [8], P[4], and P[6] are the most common human RVA G and P types globally, with G1P[8], G2P[4], G3P[8], G4P[8], G9P[8], and G12P[8] being the most commonly detected G and P genotype combinations. However, the distribution and prevalence of these common strains vary geographically and temporally [5, 16].

Currently, a whole-genome-based classification system is commonly used when studying RVA evolution and reassortment [6, 7]. In this system, all 11 RNA segments (VP7-VP4-VP6-VP1-VP2-VP3-NSP1-NSP2-NSP3-NSP4-NSP5) are assigned different genotype constellations (Gx-P[x]-Ix-Rx-Cx-Mx-Ax-Nx-Tx-Ex-Hx), where x indicates the genotype number. Most human RVAs are classified into three genotype constellations, named Wa-like, DS-1-like, and AU-1-like, and of these, AU-like strains are the least commonly detected in humans. The Wa-like genotype constellation is commonly associated with globally common strains, including G1, G3, G4, G9, G12, and P[8], while the DS-1-like constellation is commonly associated with G2 and P[4] strains [6, 26].

Epidemiological surveillance of RVAs in children with acute diarrhea at the Affiliated Children’s Hospital of Capital Institute of Pediatrics in Beijing (a level-3, grade-A, baby-friendly, and standardized services hospital) had been conducted since 1991 by our research group. In 1994, Qian et al. identified the first G9 rotavirus in mainland China, t203, in an infant with acute gastroenteritis in Beijing [8]. This was later assigned to VP7 evolutionary lineage VI, together with strains detected in Japan (K-1 and 99-TK2082) from sporadic cases in 1994-2002 as well as porcine G9 RVAs [17]. Interestingly, it was observed that t203-like G9 rotaviruses (tentatively designated as “subtype G9-VI” in our study) re-emerged in 2010 and sharply prevailed over previous major circulating G genotypes (G1-G3) and the globally common G9 (subtype G9-III) genotype during our 2011–2012 surveillance [9].

Vaccination is the most effective measure for reducing the burden of acute gastroenteritis caused by RVAs. In China, only one domestic Lanzhou lamb rotavirus (LLR) vaccine had been licensed (since 2000) before the approval of RV5 (RotaTeq) by the China Food and Drug Administration in 2018 [10]. G9 rotavirus strains are not included in the LLR and RotaTeq vaccines. It is important to gain insight into the epidemiology, evolution, and transmission shift of G9-VI rotaviruses compared to the globally common G9-III strain before considering the implementation of mass vaccination in China. In view of this, we conducted follow-up surveillance of circulating RVAs in children in Beijing for four years (2014-2017) and performed genomic analysis of the circulating G9 rotaviruses from 2010 to 2017.

## Materials and methods

### Specimens

Stool samples were collected from June 1, 2014, to December 31, 2017, which encompassed 3.5 rotavirus circulation seasons (defined as the 12-month period from June 1 of a calendar year until the end of May of the following year). All specimens were collected from pediatric outpatients younger than 15 years of age who underwent routine clinical stool tests for acute gastroenteritis, including microscopy, occult blood detection, and rotavirus detection (Colloidal Gold Device). Acute gastroenteritis was defined as at least three episodes of watery or looser-than-normal stools within a 24-h period. After routine clinical examination, the remaining samples (if sufficient material was present) were sent to our laboratory and stored at -80 °C for further analysis.

### PAGE, RT-PCR, and dot-blot hybridization for G/P genotyping

Viral dsRNA was extracted from stool specimens using TRIzol Reagent (Invitrogen, USA) according to the manufacturer’s instructions. The total recovered RNA was resuspended in 25 µl of RNase-free water, and 5 µl of this mixture was used to screen for the presence of full-length RVA nucleic acids by polyacrylamide gel electrophoresis (PAGE) and silver staining as described previously [11]. The VP7 and VP4 (VP8*) gene segments from rotavirus-positive specimens that were identified by PAGE were amplified by RT-PCR using AMV reverse transcriptase (Promega, USA) and Invitrogen Platinum Taq DNA polymerase (Thermo Fisher Scientific, USA) as described previously [12, 13]. Amplicons corresponding to the VP7 and VP4 genes were genotyped for the five most common RVA G genotypes (G1 to G4 and G9) and the three most common RVA P genotypes (P[8], P[4], and P[6]), and the G9 and P[8] amplicons were further genotyped into the G9-III, G9-VI, P[8]a, and P[8]b subtypes using dot-blot hybridization methods developed by our laboratory, which have been described in detail previously [9, 14].

### Sequence sequencing and analysis

To further investigate the molecular evolution of G9-VI rotaviruses and the mechanism underlying their selective advantage over the globally common G9-III subtype, 12 representative G9 RVA strains (including 10 G9-VI and two G9-III strains in combination with P[8]a, P[8]b, or P[6] VP4 types identified in this and previous surveillance studies from 2010 to 2017) were randomly selected for full-genome sequencing. Eleven gene segments of each representative G9 strain were amplified using RT-PCR and specific primers as described previously [6, 15]. Some of these primers were modified with degenerate bases based on the results of preliminary sequencing and alignment with sequences of circulating strains available in the GenBank database (http://www.ncbi.nlm.nih.gov/genbank/) (data not shown). The PCR products were purified using a QIAquick PCR Purification Kit (QIAGEN, Hilden, Germany) according to the manufacturer’s instructions and sent to SinoGenoMax (Beijing, China) for sequencing. The resulting sequences were analyzed using the Basic Local Alignment Search Tool (BLAST) at NCBI, and their genotypes were determined using the online Rotavirus A Genotyping Tool (version 0.1; https://www.rivm.nl/mpf/typingtool/rotavirusa/). Multiple sequence alignments were performed using ClustalW (http://www.clustal.org/clustal2/). Phylogenetic analysis was performed using MEGA 6.06 (http://www.megasoftware.net/) software, using the neighbor-joining algorithm. Bootstrapping was performed with 1000 replicates. It should be noted that the first re-emerging G9-VI rotavirus, BJ-Q94, which was identified in November 2010 and whose VP7 gene sequence was deposited in the NCBI database (accession number KF673478), was excluded from further genomic analysis in this study due to an insufficient amount of fecal material in the sample.

### Nucleotide sequence accession numbers

The full genomic nucleotide sequences of 12 representative G9 strains were deposited in the NCBI GenBank database under the accession numbers KF673477, KF673479, KF673483, KF673489, and ON563286-ON563413.

### Statistical analysis

SPSS 19.0 (IBM, USA) was used to analyze gender differences in rotavirus infection rates, using the chi-square test. *P* < 0.05 was considered statistically significant.

## Results

### Molecular epidemiology of circulating rotaviruses

**Fig. 1:**
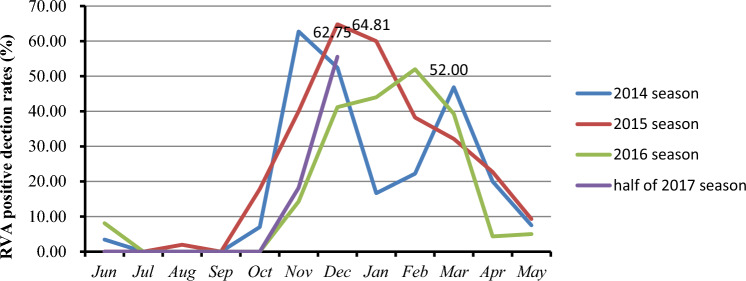
Distribution and seasonal trends of RVAs determined by analyzing the rotavirus detection rates according to month from 2014-2017. A rotavirus season was defined as a 12-month period starting from 1 June of a calendar year until the end of May of the following year

From June 2014 to December 2017, 1681 stool specimens were collected from pediatric outpatients with diarrhea, 306 of which (18.3%, 306/1681) tested positive for RVA by PAGE. The monthly RVA detection rate varied from 0 to 64.8%. Almost no RVAs circulated from July to September (summer), but RVA cases emerged from October (2014-2015) or November (2016-2017), quickly increased to a peak period lasting until March of the following year (spring), and then circulated at a low level until June (Fig. 1). Interestingly, the peak time of RVA infection in each season was delayed from 2014 to 2017, with the highest frequency of positive RVA results occurring in November (62.7%) in 2014, December (64.8%) in 2015, and February (52%) of the following year in the 2016 season (Fig. 1).

Among the RVA-positive specimens in this study, 31.4% (96/306) of infections occurred in children aged 6-11 months, while 48.7% (149/306) occurred in those aged 12-23 months, followed by the 2- to 3-year-old (8.2%) and <6 months (5.9%) group, with only a few infections occurring in children over 5 years of age (1.6%, 5/306). Therefore, most RVA-infected children were between 6 and 23 months old (80.1%), which is consistent with our previous surveillance results. RVA infections were more common in boys than in girls in this study, with a sex ratio (male/female, 195/111) of 1.76; however, this difference in distribution was not statistically significant (*p* = 0.65).

Of the 306 RVA-positive samples identified by PAGE, 305 contained an amplifiable VP7 gene and were G-genotyped, while 301 were amplified by RT-PCR for the VP4 and P genotypes. Gene segment degradation and/or the presence of substances in the fecal total RNA that inhibited amplification led to unsuccessful amplification for VP7 and/or VP4 genes in some samples.

**Fig. 2:**
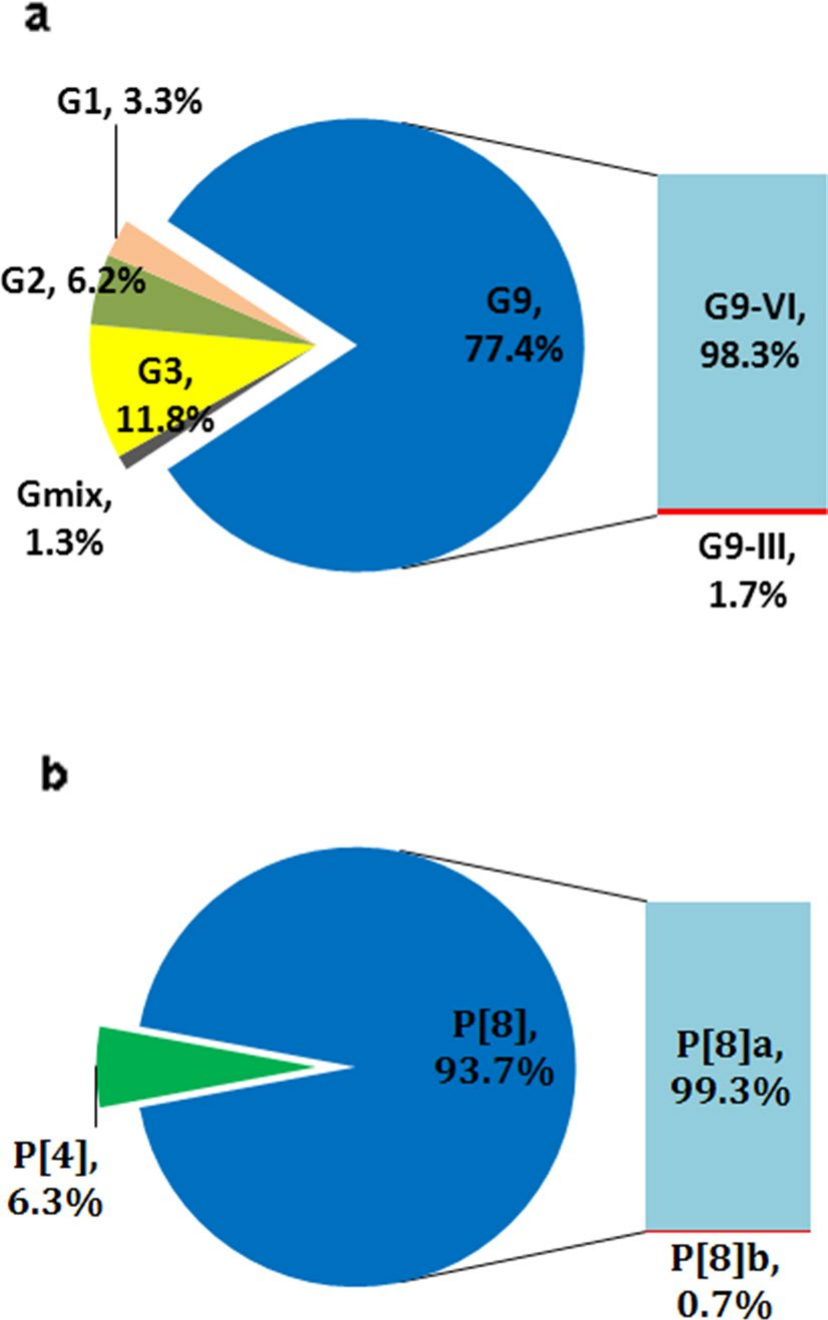
Frequency of RVA G (a) and P (b) genotypes/subgenotypes detected in clinical samples from June 2014 to December 2017 in Beijing. Each sector represents the relative distribution of an RVA G or P genotype/subgenotype based on amplicons of VP7 (n = 305) and VP4 (n = 301) genes and genotyping by dot-blot hybridization, respectively

Compared with the previous G9 prevalence (43.5%) identified in our 2011-2012 surveillance [10], the prevalence of G9 in this study sharply increased to 77.4% (236/305), followed by G3 (11.8%), G2 (6.2%), and G1 (3.3%). Mixed G genotypes were detected with a prevalence of 1.3% (Fig. 2a). During the 2014 and 2015 seasons, G9 circulated at a prevalence of 87.6% and 83.3%, respectively, but this suddenly decreased to 56.1% in the following 2016 season before increasing again to 64.3% during the 2017 season. Notably, the majority (98.3%) of these circulating G9 RVAs still belonged to the G9-VI subtype, whereas subtype G9-III, which is the most common G9 strain globally, accounted for only a small proportion (1.7%) of cases.

VP4 genotyping revealed that the most prevalent P genotype was P[8] (93.7%), followed by P[4] (6.3%), and the P[6] genotype was not detected in this study (Fig. 2b). Interestingly, most of the P[8] strains still belonged to the globally common P[8]a subtype, whereas P[8]b-subtype cases were rare (0.7%) during our study period (Fig. 2b). In accordance with the G genotype distribution, G9P[8] was the most prevalent G-P combination (77%), followed by G3P[8](12%), G2P[4] (6.3%), and G1P[8] (3.3%).

### Genome sequence analysis of representative G9 rotaviruses

Complete coding sequences (CDSs) for each gene were obtained from all 12 representative G9 strains. Interestingly, all of the G9-VI and G9-III-subtype rotaviruses were combined with VP4 genotype P[8]a, P[8]b, or P[6], and all of the representative G9 strains in this study exhibited a common Wa-like I1-R1-C1-M1-A1-N1-T1-E1-H1 genogroup constellation. A comparison of the genomic configuration of the 12 G9 RVA strains with those of other selected human reference G9 rotaviruses whose whole-genome sequences were available in the GenBank database is summarized in Table 1, in which subtype P[8]a is written as “P[8]” in accordance with traditional nomenclature.

**Table 1:** Genotype constellations of the 12 G9 RVA strains from this study (bold) and selected G9 reference strains from the GenBank database

Strain name	Genotype/sub-genotype										
	VP7	VP4	VP6	VP1	VP2	VP3	NSP1	NSP2	NSP3	NSP4	NSP5
RVA/Human-tc/USA/WI61/1983/G9P1A[8]	G9	P[8]	I1	R1	C1	M1	A1	N1	T1	E1	H1
**RVA/Human-wt/CHN/BJ-F1565/2017/G9P[8]**	**G9-III**	**P[8]**	**I1**	**R1**	**C1**	**M1**	**A1**	**N1**	**T1**	**E1**	**H1**
**RVA/Human-wt/CHN/BJ-Q33/2010/G9P[8]b**	**G9-III**	**P[8]b**	**I1**	**R1**	**C1**	**M1**	**A1**	**N1**	**T1**	**E1**	**H1**
**RVA/Human-wt/CHN/BJ-CR8090/2012/G9P[8]b**	**G9-VI**	**P[8]b**	**I1**	**R1**	**C1**	**M1**	**A1**	**N1**	**T1**	**E1**	**H1**
**RVA/Human-wt/CHN/BJ-Q322/2011/G9P[8]**	**G9-VI**	**P[8]**	**I1**	**R1**	**C1**	**M1**	**A1**	**N1**	**T1**	**E1**	**H1**
**RVA/Human-wt/CHN/BJ-Q1087/2012/G9P[8]**	**G9-VI**	**P[8]**	**I1**	**R1**	**C1**	**M1**	**A1**	**N1**	**T1**	**E1**	**H1**
**RVA/Human-wt/CHN/BJ-CR9205/2013/G9P[8]**	**G9-VI**	**P[8]**	**I1**	**R1**	**C1**	**M1**	**A1**	**N1**	**T1**	**E1**	**H1**
**RVA/Human-wt/CHN/BJ-F495/2014/G9P[8]**	**G9-VI**	**P[8]**	**I1**	**R1**	**C1**	**M1**	**A1**	**N1**	**T1**	**E1**	**H1**
**RVA/Human-wt/CHN/BJ-F913/2015/G9P[8]**	**G9-VI**	**P[8]**	**I1**	**R1**	**C1**	**M1**	**A1**	**N1**	**T1**	**E1**	**H1**
**RVA/Human-wt/CHN/BJ-F1435/2016/G9P[8]**	**G9-VI**	**P[8]**	**I1**	**R1**	**C1**	**M1**	**A1**	**N1**	**T1**	**E1**	**H1**
**RVA/Human-wt/CHN/BJ-F1463/2016/G9P[8]**	**G9-VI**	**P[8]**	**I1**	**R1**	**C1**	**M1**	**A1**	**N1**	**T1**	**E1**	**H1**
**RVA/Human-wt/CHN/BJ-J1/2017/G9P[8]**	**G9-VI**	**P[8]**	**I1**	**R1**	**C1**	**M1**	**A1**	**N1**	**T1**	**E1**	**H1**
**RVA/Human-wt/CHN/BJ-CR7927/2012/G9P[6]**	**G9-VI**	**P[6]**	**I1**	**R1**	**C1**	**M1**	**A1**	**N1**	**T1**	**E1**	**H1**
RVA/Human-wt/GHA/DC949/2010/G9P[8]b	G9	P[8]b	I1	R1	C1	M1	A1	N1	T1	E1	H1
RVA/Human-wt/TGO/MRC-DPRU5123/2010/G9P[8]	G9	P[8]	I1	R1	C1	M1	A1	N1	T1	E1	H1
RVA/Human-wt/CHN/JS2011/2011/G9P[8]	G9	P[8]	I1	R1	C1	M1	A1	N1	T1	E1	H1
RVA/Human-wt/CHN/km15118/2015/G9P[8]	G9	P[8]	I1	R1	C1	M1	A1	N1	T1	E1	H1
RVA/Human-wt/JPN/To14-32/2014/G9P[8]	G9	P[8]	I1	R1	C1	M1	A1	N1	T1	E1	H1
RVA/Human-wt/ZWE/MRC-DPRU1102/2012/G9P[8]	G9	P[8]	I1	R1	C1	M1	A1	N1	T1	E1	H1
RVA/Human-wt/USA/VU12-13-101/2013/G9P[8]	G9	P[8]	I1	R1	C1	M1	A1	N1	T1	E1	H1
RVA/Human-wt/CHN/km15035/2015//G9P[8]	G9	P[8]	I1	R1	C1	M1	A1	N1	T1	E1	H1
RVA/Human-wt/CHN/JS2016/2016/G9P[8]	G9	P[8]	I1	R1	C1	M1	A1	N1	T1	E1	H1
RVA/Human-wt/JPN/UR14-21/2014/G9P[8]	G9	P[8]	I1	R1	C1	M1	A1	N1	T1	E1	H1

### The VP7 gene

**Fig. 3:**
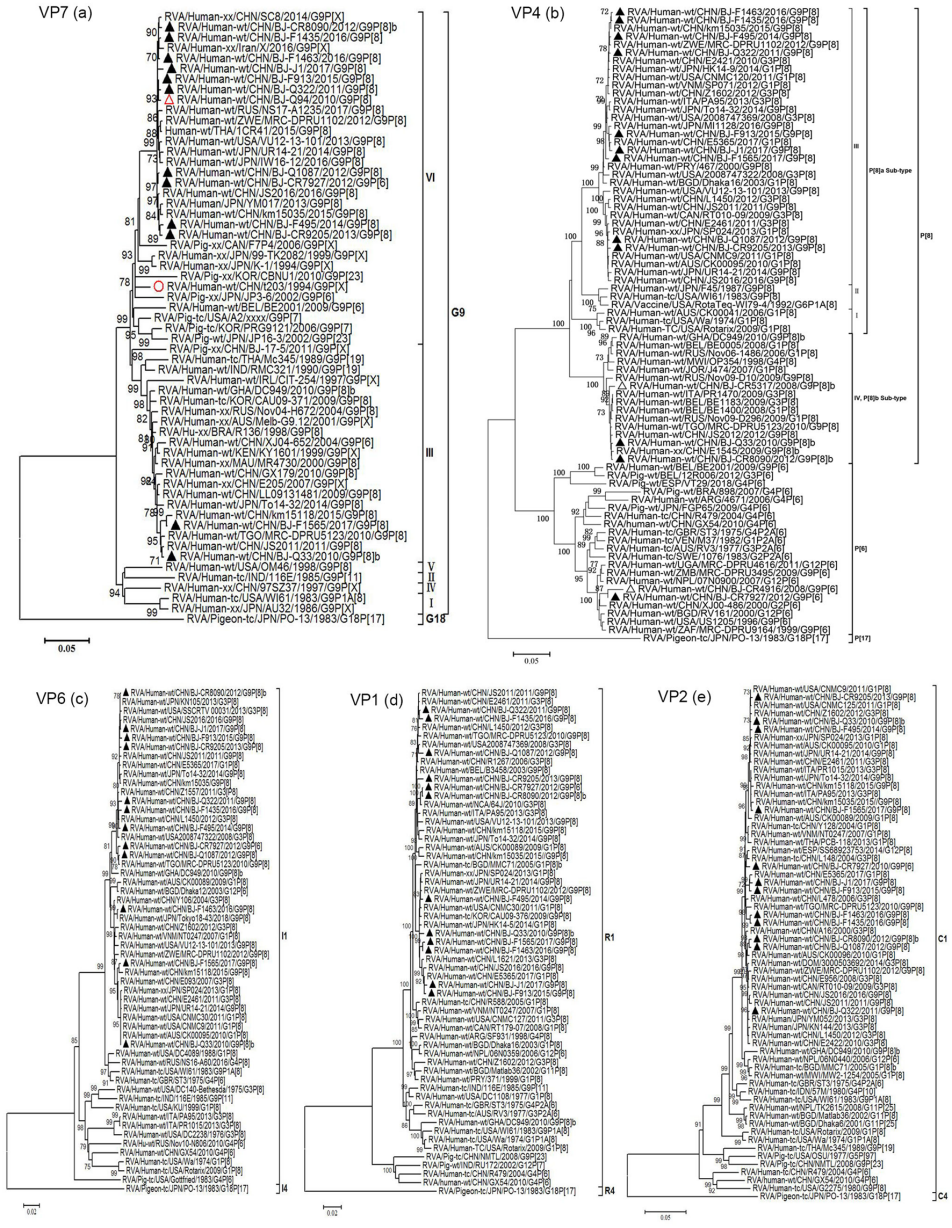

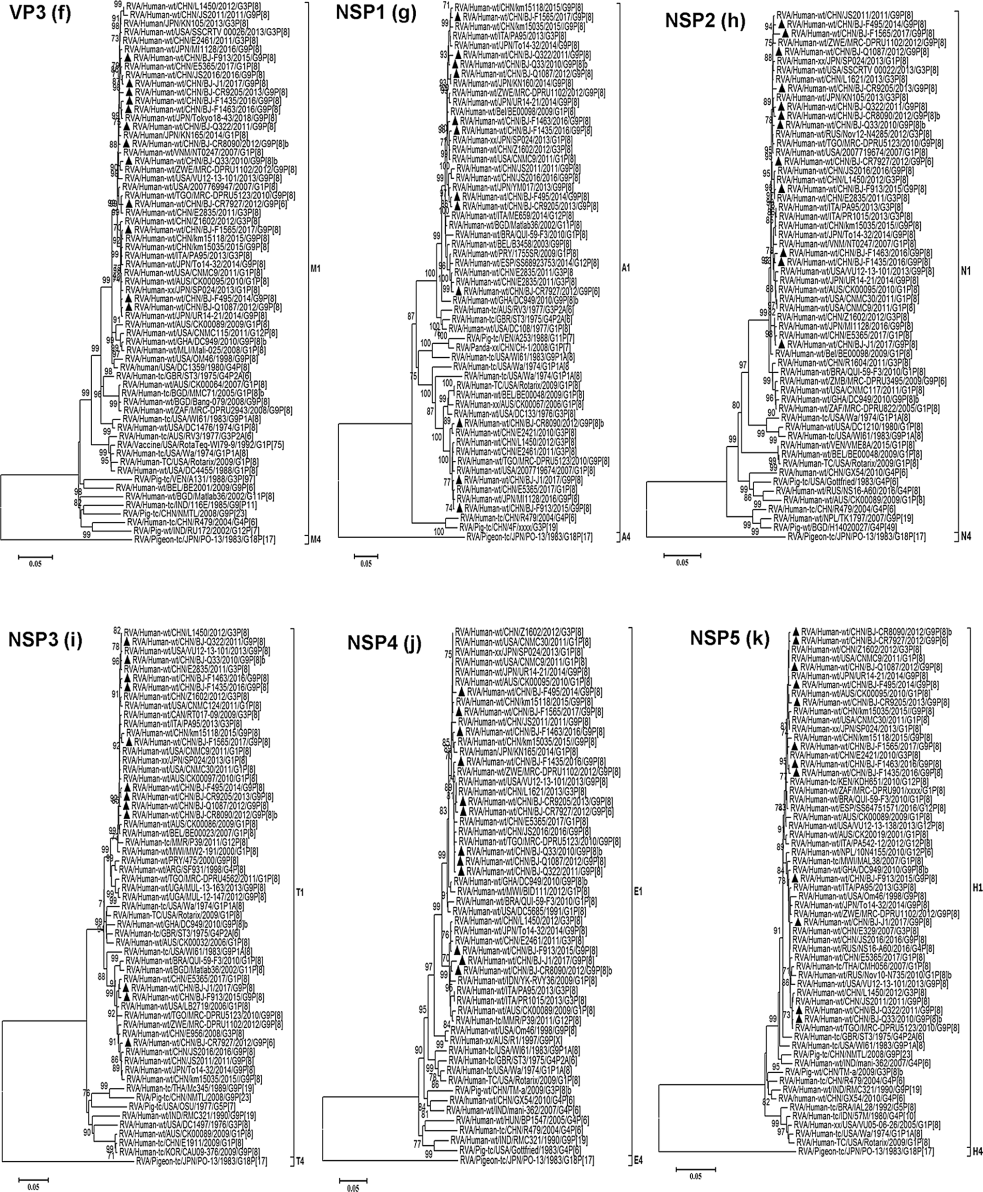
Phylogenetic trees based on individual genes of RVAs. The phylogenetic trees were constructed based on the nucleotide sequences of the RVA VP7 (a), VP4 (b), VP6 (c), VP1 (d),VP2 (e), VP3 (f), NSP1 (g), NSP2 (h), NSP3 (i), NSP4 (j), and NSP5 (k) gene segments of 12 G9 strains from this study (indicated by solid triangles) and those of reference strains obtained from the GenBank database. The first G9-VI strain, t203, and the first detected re-emergent G9-VI strain, BJ-Q94, in China are indicated by a red open circle and a triangle, respectively, in the VP7 tree. The neighbor-joining method with 1000 bootstrap replicates was used. Bootstrap values lower 70% are not shown. The genotypes and/or lineages within a genotype are indicated at the right. The scale bar shows the genetic distance. The sequence of the corresponding gene of strain RVA/Pigeon-tc/JPN/PO-13/1983/G18P[17] was included as an outgroup

Phylogenetically, 10 out of the 12 G9 strains genotyped as G9-VI in this study clustered closely with the BJ-Q94 strain, which was identified in our laboratory in 2010, as well as with contemporary emerging human G9 strains (with sequences published previously or submitted directly to the GenBank database) from Zimbabwe (MRC-DPRU1102/2012), Japan (YM017/2013 and UR14-21/2014), USA (VU12-13-101/2013), China (SC8/2014, km15035/2015, and JS2016/2016), Thailand (1CR41/2015), Russia (NS17-A1235/2017), and Iran (GenBank accession number MH824633). These strains formed a monophyletic subcluster that was distinct from early human G9-VI rotaviruses identified in China (t203/1994) and Japan (K-1/1994 and TK2091/1999), as well as many porcine strains from around the world within the VP7 gene evolutionary lineage VI (Fig. 3a). The other two G9 strains from this study belonged to VP7 lineage III, which includes common globally circulating human G9 rotaviruses (Fig. 3a). All of the representative G9 strains in this study, whether G9-VI or G9-III, clustered separately from the G9 prototype strain WI61 and other early human G9 strains (e.g., 116E, 97SZ37, and om46), within different VP7 lineages.

**Fig. 4:**
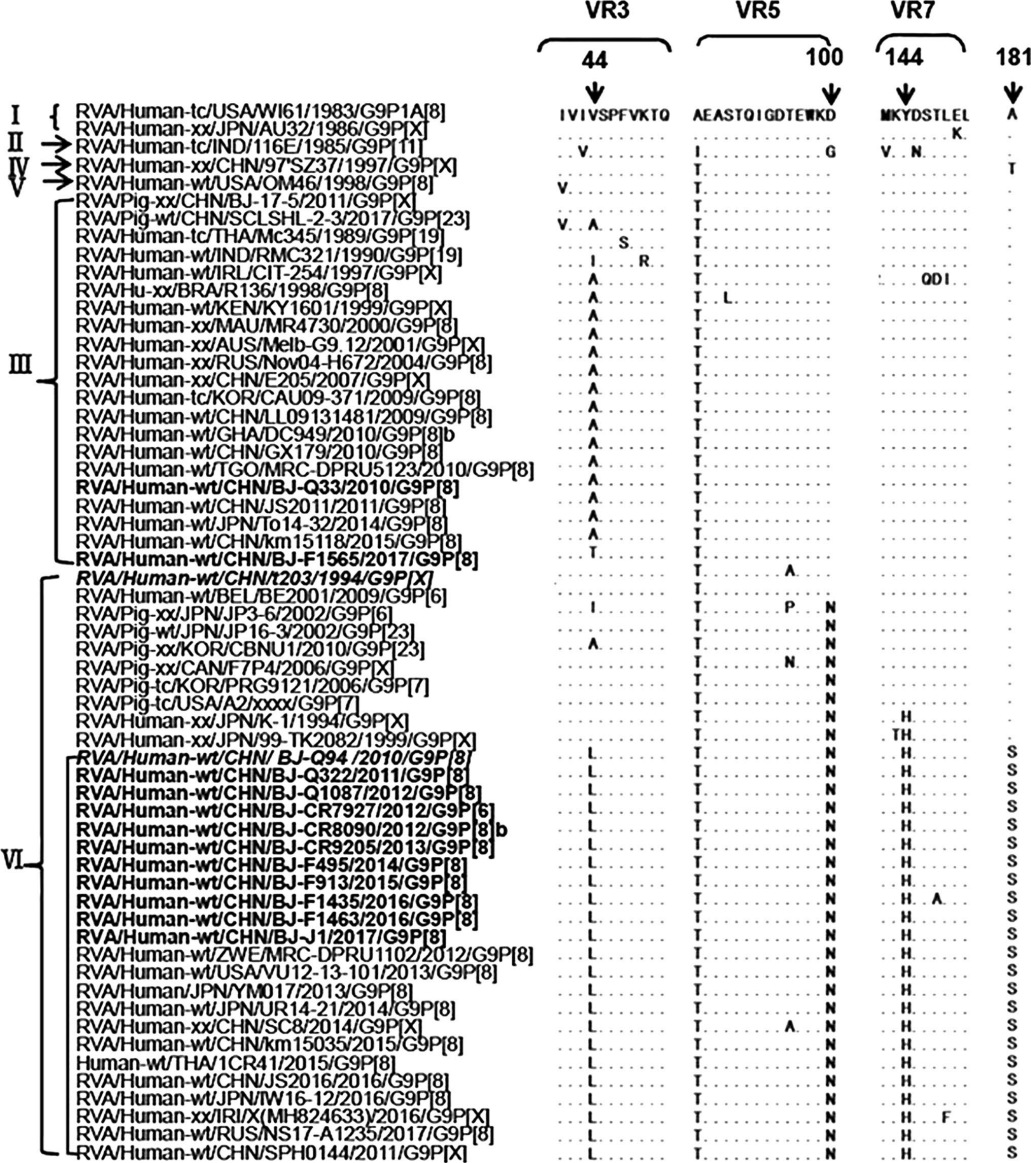
Alignment of the deduced amino acid sequences of VP7 of G9 rotaviruses within different evolutionary lineages (I-VI) in this study. The 12 representative G9 strains from this study are shown in bold, and the first G9-VI strain, t203, and the first detected re-emergent G9-VI strain, BJ-Q94, in China, identified previously in our laboratory, are shown in italics. Amino acid residues that are identical to the corresponding residue of classical G9 strain RVA/Human-tc/USA/WI61/1983/G9P1A[8] are shown as dots. Four specific amino acid substitutions at positions 44, 100, 144, and 181 between G9-VI and other G9 rotaviruses within other different lineages, are indicated by a downward arrow, of which aa 44, 100, and 144 are located in the variable regions VR3, VR5, and VR7, respectively

Multiple alignment analysis was performed based on the VP7 genes as well as the deduced amino acid sequences of the encoded proteins of the G9 strains in the phylogenetic tree. The G9 strains in this study shared 91.8%-99.9% nucleotide sequence identity and 94.8%-100% amino acid sequence identity. The VP7 genes of the currently emerging G9-VI RVAs exhibited high nucleotide (98.5%-99.9%) and amino acid (98.5%-100%) sequence identity to each other but lower nucleotide (92.2%-95.6%) and amino acid (95.1%-97.6%) sequence identity to porcine and early human rotaviruses within lineage VI. Interestingly, four characteristic and conserved amino acids at positions 44(L), 100(N), 144(H), and 181(S) were observed in all of the recently emerging G9-VI rotaviruses included in the analysis when compared with globally common G9-III and other studied G9 strains within different lineages; specifically, D/G100N and Y144H were also present in some early human (e.g., K-1 and TK2091) and/or porcine (originating from Japan, China, the USA, and Canada) G9-VI strains (Fig. 4). In addition, synonymous codons were found at amino acid positions 55, 96, 126, 196, 220, 222, 227, 254, and 302 of the currently circulating G9-VI and globally common G9-III RVAs. The corresponding nucleotide bases at the third position of these codons were more specific and conserved among recently emerging G9-VI RVAs (data not shown).

### The VP4 gene

Phylogenetic analysis of the VP4 gene revealed that all P[8]a-subtype strains in this study clustered in the P[8]-III lineage, alongside contemporary P[8] strains that are spreading worldwide. Two P[8]b-subtype strains in this study (BJ-Q33 and BJ-CR8090, identified in 2010 and 2012, respectively) clustered closely with P[8]b strains from China, Togo, and Russia within lineage IV (Fig. 3b). In contrast, BJ-Q33 and BJ-CR8090 clustered separately with the P[8]b prototype strain OP354, isolated in Malawi in 1998, also within P[8] lineage IV. According to our sequence analysis of the VP4 genes, the P[8]a-subtype strain in this study exhibited high nucleotide (96.8%-99.7%) and amino acid (98.1%-99.9%) sequence identity to strains within the same lineage (III) but lower nucleotide (88.6%-88.9%) and amino acid (92.5%-93.2%) sequence identity to P[8]b-subtype strains.

The P[6] strain in this study (BJ-CR7927/2012) was closely related to the BJ-CR4916 strain (accession number GU937090, identified in our laboratory in 2008) and common human P[6] strains detected globally, but was distantly related to early human P[6]-genotype strains (e.g., ST3, RV3, and 1076) (Fig. 3b).

### Other structural protein (VP6 and VP1-VP3) and non-structural protein (NSP1-NSP5) genes

The remaining nine genes of each of the 12 representative G9 strains in this study possessed the same Wa-like genomic backbone (I1-R1-C1-M1-A1-N1-T1-E1-H1). Sequence analysis showed that VP6, VP1-VP3, NSP2, and NSP5 exhibited the highest nucleotide sequence conservation, with sequence differences ranging from 0.1 to 2.9%. In contrast, the NSP3 and NSP4 genes exhibited slightly lower sequence similarity, with 93.3-99% and 95.5-99.9% nucleotide sequence identity and 95.8-100% and 95.5-100% amino acid sequence identity, respectively. The NSP1 gene showed the most sequence diversity among the 12 G9 strains, with 84.3-99.6% nucleotide sequence identity and 82.2-99.8% amino acid sequence identity.

Phylogenetic analysis based on the VP6, VP1-VP3, and NSP1-NSP5 gene sequences revealed that the 12 studied G9 strains, whether G9-III or G9-VI, combined with P[8]a, P[8]b, or P[6], all clustered closely together with contemporary human RVA G9P[8], G3P[8], G1P[8], G12P[8], and/or G12P[6] reference strains. These were mostly detected in neighboring geographical areas such as China and Japan and had similar Wa-like genotype backbones. Although their corresponding cognate genes showed a high degree of sequence similarity, they were not consistently distributed into specific phylogenetic lineages or clusters (Fig. 3c-k). For example, strain BJ-CR8090 clustered closely with strains BJ-F913 and BJ-J1 and was closely related to the Chinese G3P[8] strain E2421 based on its NSP1 gene (Fig. 3g). However, the NSP3 gene of BJ-CR8090 was closely related to the American G1P[8] strain CNMC9 and more distantly related to F913 and J1 in different evolutionary lineages (Fig. 3i).

## Discussion

Since the mid-1990s, G9 has emerged worldwide and is now recognized as the fifth global common G genotype [5, 16, 17]. Phylogenetically, G9 rotaviruses are commonly classified into six evolutionary lineages based on the VP7 gene [17]. The globally common G9 RVAs belong to lineage III (G9-III), while lineage VI (G9-VI) includes unusual and sporadically occurring human rotaviruses that were identified in China (t203) and Japan during 1994-2002, as well as porcine G9 rotaviruses [17]. In our previous surveillance, we found that G9 rotaviruses within VP7 lineage VI had re-emerged (tentatively designated as the G9-VI subtype) in 2010 and then rapidly prevailed over circulating G9-III and other common rotavirus genotypes over the next two years in children in Beijing [9]. To obtain insight into the epidemiology, evolution, and transmission advantages of G9-VI rotaviruses, RVA surveillance follow-up was continued for four years (2014-2017), and full-genome analysis of 12 selected representative G9 strains (identified from 2010 to 2017) were conducted.

The results indicated that over the follow-up period (3.5 seasons), G9 was still the most predominant genotype (77.4%) and had increased sharply in prevalence (previously 43.5%). The prevalence of G3 (30.5%), G1 (12.2%), and G2 (11.5%) rotaviruses in the previous 2011-2012 surveillance period [9] had further decreased (11.8%, 3.3%, and 6.2%, respectively) over this surveillance period. Interestingly, among the circulating G9 RVAs, subtype G9-VI remained the most dominant member, accounting for 98.3% of cases, with little difference from prior surveillance (96.5%). The globally common circulating G9 rotavirus (subtype G9-III) accounted for only a small proportion (1.7%). This suggested that G9-VI subtype rotaviruses remained predominant and had an absolute transmission advantage over the globally common G9-III in this 2014-2017 surveillance of children in Beijing.

Based on data from our previous surveillances, G3, G1, and G2 were the major circulating G genotypes in children before 2011, while P[8] and P[4] were the most common P genotypes [9, 14]. Although the P[6] genotype is common worldwide [5, 15, 16], we almost never detected P[6] in outpatients, except in hospitalized children in Beijing. Notably, there was no G9 circulation reported in Beijing since the first G9 strain (t203) was reported in 1994 until the 2006-2010 surveillance study, which showed that G9 re-emerged in outpatients at a low prevalence rate (9.3%). The VP7 genes of these G9 rotaviruses were closely related to globally common G9 RVAs (lineage III) [18]. Subsequently, t203-like G9 (subtype G9-VI) re-emerged in 2010 and soon replaced the previously dominant G genotypes (G1-G3) and G9-III as the predominant circulating genotype. Moreover, G9 rotaviruses (whether subtype G9-VI or G9-III), in our surveillance, mainly combined with the globally common circulating P[8] type (subtype P[8]a), and only a few combined with the P[8]b subtype or P[6] genotypes [9, 14, 18].

P[8]b-subtype RVAs (OP354-like rotaviruses, which are currently classified as a genetically distinct P[8] subtype) were first reported in Malawi (Africa) and gained attention recently due to their apparent global spread; their origin was traced by phylogeographic studies in Asia, with subsequent spread to other continents [19, 20]. However, the actual global prevalence of P[8]b (P[8]-lineage IV) remains largely unknown because it is undetectable when using commonly used P-genotyping primers, whereas gene sequencing can distinguish it from the globally prevailing P[8]a subtype (P[8]-lineage I–III) [21]. In our previous study, we first reported a rare P[8]b (BJ-CR5317/GU980591) rotavirus strain circulating in Beijing in 2008 and then developed an effective dot-blot hybridization method to identify P[8]a, P[8]b, P[4], and P[6] genotypes/sub-genotypes of the VP4 genes to investigate the prevalence of the P[8]b subtype in Beijing [14]. In the present study, the prevalence of the P[8]b subtype in outpatients was lower (0.7%, than in the previous study (2.3%) [14]. The P[8]b subtype was mainly combined with the G9 type and occasionally the G1 type, in our surveillance, however, due to the limited number of samples tested, no statistically relevant preference for a particular G genotype was identified. Nevertheless, whether this currently rare P[8]b subtype could replace the globally common P[8]a (similarly to how G9-VI replaced G9-III) and affect rotavirus epidemiology in the future will be known only through continuous monitoring.

Phylogenetically, the VP7 genes of the 10 G9-VI strains in this study closely clustered with those of the BJ-Q94 strain detected in 2010 and contemporary emerging human G9 strains. These strains collectively formed a monophyletic subcluster distinct from early human G9 strains (e.g., t203 and K-1) and porcine strains within lineage VI; nevertheless, they all clustered distantly from the globally common circulating G9-III RVAs. Interestingly, these contemporary circulating G9 rotaviruses within lineage VI, which had been published or had sequences available in the GenBank database, circulated not only in other regions (e.g., Jiangsu and Yunnan) in China but also in other countries (e.g., Japan, Zimbabwe, Thailand, the USA, Russia, and Iran) [22–25]. This demonstrates their potential for global spread. Therefore, our results indicated that G9-VI subtype RVAs in children with diarrhea might become a significant global health concern.

Genomic and phylogenetic analysis revealed that regardless of whether G9-VI or G9-III combined with P[8]a, P[8]b, or P[6], all 12 studied representative G9 strains shared a common Wa-like genotype constellation (I1-R1-C1-M1-A1-N1-T1-E1-H1). They also all clustered together with worldwide circulating human G9P[8], G3P[8], G1P[8], G12P[8], and G12P[6] RVA reference strains, without consistent evolutionary cluster distribution patterns. Unlike the P[8] genotype strains, which are mostly associated with the Wa-like constellation, the P[6] strains (both common in human and porcine RVAs) can be associated with not only Wa-like but also DS-l-like genetic backbones [15, 26, 27]. Because the P[6] genotype was less frequently detected in our surveillance, BJ-CR7927 (G9-VI, 2012) was the only genomically analyzed P[6] strain in this study. However, we did find that it was closely related to strain BJ-CR4916, which belonged to the G9-III RVAs previously identified in our laboratory in 2008 (accession numbers GU937089 and GU937090 for the VP7 and VP4 gene, respectively) and clustered with common human P[6] strains detected globally.

G9-VI and G9-III RVAs were reported worldwide at approximately the same time in the mid-1990s, and it has been suggested that human G9 and porcine G9 RVAs have a common origin [17, 28]; however, G9-III rotaviruses gained a transmission advantage in the human population and spread globally over a very short time span [16, 17]. Based on our present and previous surveillance data, during this short time, G9-VI rotaviruses adapted and developed an evolutionary advantage in Beijing, China [9]. However, since G9 strains can combine with P genotypes in many different variations, and the remaining nine genes were also not specific between these G9 strains, we were curious about what drove the current G9-VI strain to surpass G9-III in transmission in pediatric outpatients in Beijing.

To better understand the transmission shift from G9-III to G9-VI, we analyzed the nucleotide and deduced amino acid sequences of VP7 from representative G9 strains in the phylogenetic tree based on multiple alignments. To obtain more-comprehensive sequence information and investigate the molecular characteristics of the currently circulating G9-VI strains, as many sequences as possible (over 100; with complete CDSs available in the GenBank database and with high nucleotide sequence identity (>98.5%) to the currently emerging G9-VI based on BLAST; accessed on July 2, 2021) were included (data not shown). Interestingly, four characteristic and specific amino acids at positions 44(L), 100(N), 144(H), and 181(S) were observed in the current emerging G9-VI, which is different from the globally common G9-III RVAs, as well as other G9 rotaviruses within different evolutionary lineages. Of these, aa44L and aa181S were only conserved in the recently emerging G9-VI RVAs, whereas aa100N and aa144H were also found in some early human (e.g., K-1 and TK2091) and/or porcine (from Japan, China, the USA, and Canada) G9-VI RVAs. Moreover, aa 44, 100, and 144 were located in VR3, VR5 (major antigenic region A), and VR7, respectively, which belong to one of the nine previously described variable sequence regions (VR1-VR9) and/or six neutralizing epitopes (antigenic regions A–F) [1, 29].

Notably, more synonymous codon usage was observed between the currently emerging G9-VI and globally common G9-III RVAs. Synonymous sites are generally considered functionally neutral; however, researchers have recently recognized synonymous codon usage bias (SCUB) in the genomes of almost all species, which can affect gene expression and protein levels through a wide variety of mechanisms [30, 31]. Thus, whether specific amino acid substitutions at aa 44, 100, 144, and 181 or SCUB increased the protein expression levels of subtype G9-VI VP7 genes over those of G9-III RVAs and drove its predominance in Beijing over other globally common strains requires further investigation.

Since the global introduction of RVA vaccines, many studies have reported changes in the distribution and prevalence of RVA genotypes, and even seasonal and disease patterns of rotavirus infections, and whether these shifts in RVA epidemiology are driven by immune pressure from mass vaccination has become a significant global health concern [32–35]. In China, RV5 (RotaTeq) was introduced in 2018 [10], whereas LLR has been licensed since 2000. Vaccination coverage of LLR was not geographically uniform (primarily used in southern China, which had good economic conditions), and most vaccinated children received only one dose [10, 36]. Interestingly, before 2018, the major circulating RVA genotype had undergone three shifts – from G1 (before 2000) to G3 (2001-2011) and from G3 to G9 (2011-2018) [10] – including the G9 subtype shift from the globally common G9-III to the currently re-emerging G9-VI [9]. Therefore, it is not likely that vaccination contributed to these shifts in rotavirus distribution in China. Furthermore, the seasonal peak of circulating RVA was delayed in this study, which also occurred in the post-vaccination era in Belgium [34]. Therefore, rotavirus prevalence and genotype distribution in children might not only be affected by vaccine immune pressure but also by herd immune pressure.

In conclusion, rotavirus subtype G9-VI continued to be the predominant subtype and gained an absolute transmission advantage over previously predominant G genotypes and globally common G9-III RVAs during the 2014-2017 surveillance period in children in Beijing. G9-VI-subtype rotaviruses are capable of spreading globally and may become a great health concern, affecting children worldwide. However, whether specific amino acid substitutions and synonymous codon usage bias promoted strong expression of VP7 genes in G9-VI and drove its circulation requires further investigation. Furthermore, determining whether the currently rare P[8]b subtype will become a major circulating P-type (superseding the presently common P[8]a) in the future and whether the circulation of rotaviruses with nontraditional genotypes will impact vaccine efficacy will require large-scale, long-term RVA surveillance worldwide.

### Supplementary Information

Below is the link to the electronic supplementary material.

Supplementary file1 (RAR 187 KB)

Supplementary file2 (DOCX 1437 KB)

## Data Availability

The nucleotide sequences of all 12 representative G9 strains in this study were deposited in the GenBank database under accession numbers KF673477, KF673479, KF673483, KF673489 and ON563286- ON563413.
