# Supplementary material for: Prevalence and genomic analysis of t203-like G9 (G9-VI) rotaviruses circulating in children with gastroenteritis in Beijing, China
Source: Arch Virol. 2023 Sep 27;168(10):257. doi: 10.1007/s00705-023-05860-0 (PMC10533636; doi:10.1007/s00705-023-05860-0)
Supplement: Supplementary file 2 — Supplementary file2 (DOCX 1437 KB) [file 705_2023_5860_MOESM2_ESM.docx]

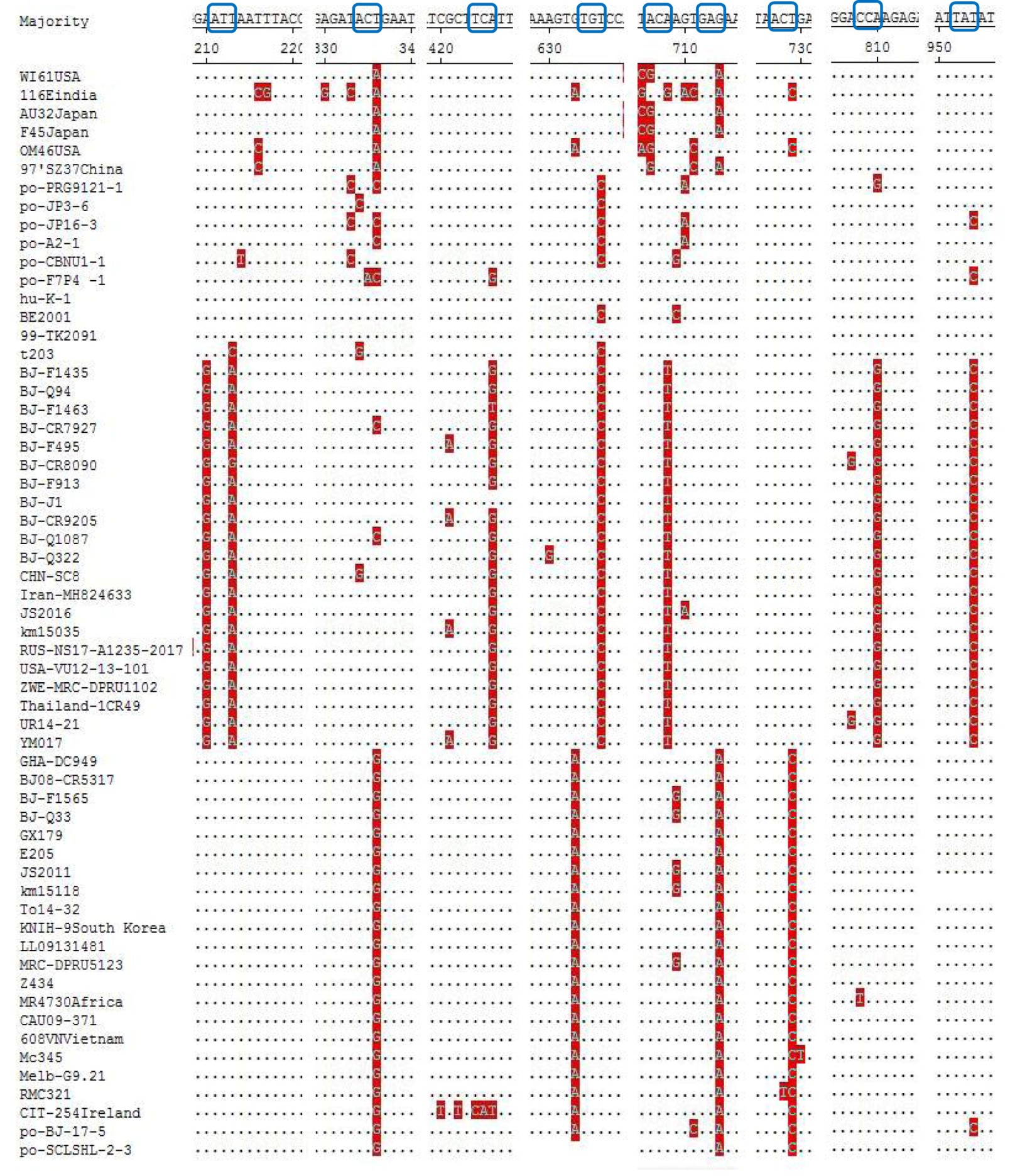


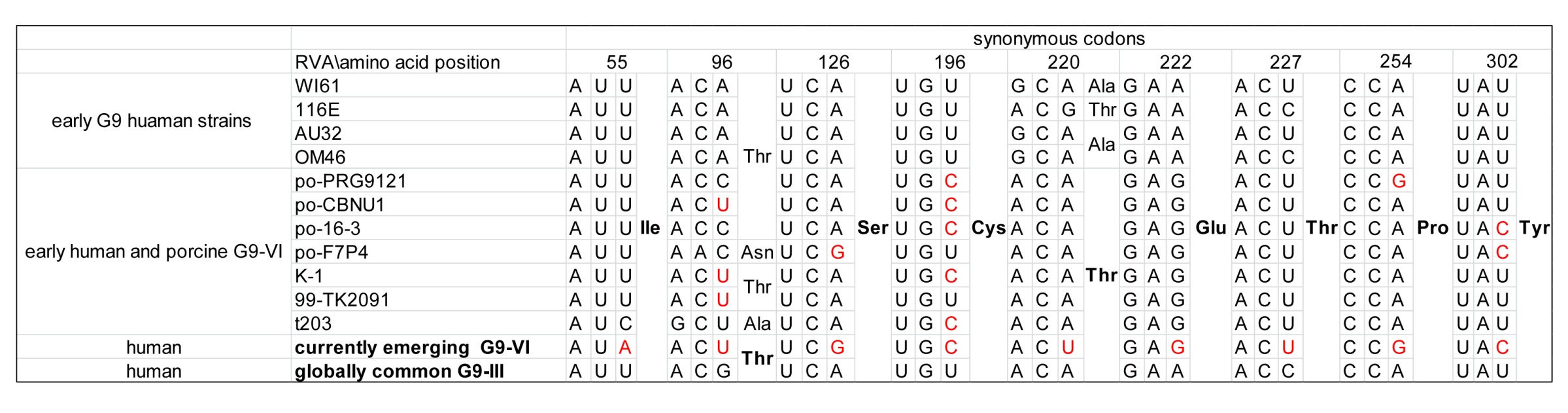


1A

Suppl. Figure 1. Synonymous codons at amino acid positions 55, 96, 126, 196, 220, 222, 227, 254 and 302 between the currently circulating G9-VI and globally common G9-III RVAs

1B
